# Gain–of–Function Genetic Models to Study FSH Action

**DOI:** 10.3389/fendo.2019.00028

**Published:** 2019-02-07

**Authors:** Rosemary McDonald, Carolyn Sadler, T. Rajendra Kumar

**Affiliations:** ^1^Division of Reproductive Sciences, Department of Obstetrics and Gynecology, University of Colorado Anschutz Medical Campus, Aurora, IL, United States; ^2^Integrated Physiology Graduate Program, University of Colorado Anschutz Medical Campus Aurora, IL, United States; ^3^Division of Reproductive Endocrinology and Infertility, Department of Obstetrics and Gynecology, University of Colorado Anschutz Medical Campus Aurora, IL, United States

**Keywords:** pituitary, follicle-stimulating hormone, transgenic mice, testis, ovary

## Abstract

Follicle–stimulating hormone (FSH) is a pituitary-derived gonadotropin that plays key roles in male and female reproduction. The physiology and biochemistry of FSH have been extensively studied for many years. Beginning in the early 1990s, coincident with advances in the then emerging transgenic animal technology, and continuing till today, several gain-of-function (GOF) models have been developed to understand FSH homeostasis in a physiological context. Our group and others have generated a number of FSH ligand and receptor GOF mouse models. An FSH GOF model when combined with *Fshb* null mice provides a powerful genetic rescue platform. In this chapter, we discuss different GOF models for FSH synthesis, secretion and action and describe additional novel genetic models that could be developed in the future to further refine the existing models.

## Introduction

Follicle–Stimulating Hormone (FSH) is a gonadotropin synthesized in gonadotropes of the anterior pituitary gland. FSH is a heterodimeric glycoprotein, consisting of two distinct α- and β (FSHβ) subunits (1–4). The α-subunit is structurally identical to both gonadotropins–luteinizing hormone (LH), and chorionic gonadotropin (CG) as well as thyroid-stimulating hormone (TSH). FSH subunits are encoded by distinct genes. The FSHβ subunit is unique and confers the biological specificity for FSH functions (1–4). The β-subunits exist in comparatively lower amounts within the pituitary than their corresponding α-subunit. FSH subunits are synthesized and assembled non-covalently in gonadotropes (1–4). Non-covalent linkage of the subunits allows for easy separation and hybridization, yet free α and β-subunits are typically expressed by other tissues under a variety of pathological conditions (1–4).

FSH signaling in the hypothalamic-pituitary-gonadal axis (HPG) regulates critical reproductive functions such as steroidogenesis and gametogenesis. In males, FSH contributes to spermatogenesis and testicular development by binding to Sertoli cells and regulating their development and differentiation (5–7). In females, FSH contributes to ovarian follicular development by upregulating aromatase expression in granulosa cells, which results in increased estrogen production. Increased estrogen synthesis is required for normal follicular growth (8).

FSH and LH are synthesized and released in response to gonadotropin-releasing hormone (GnRH) secreted from the hypothalamus. GnRH binds to GnRH- receptors on pituitary gonadotropes (4, 9). Differing GnRH release frequencies favor either FSH or LH synthesis as gonadotropes are sensitive to patterns of GnRH stimulation ad respond by altering hormone-specific subunit gene transcription. LH is secreted in a regulated, pulsatile fashion in response to increased GnRH pulse frequency, whereas FSH is released is mostly constitutive and responds to decreased GnRH pulse frequency (10, 11).

FSH synthesis and release are also regulated by several other proteins, such as follistatin, inhibin, and activin (12–17). Activin exerts positive effects on FSH by stimulating transcription, biosynthesis, and ultimately secretion as well as stimulating GnRH receptor gene expression. In rodents, after activin binds to gonadotrope membrane activin receptors, transcription factors, such as Smad4 are recruited and directly interact with the FSHβ gene promoter to upregulate its expression (15, 18). Another important transcription factor in gonadotropin regulation is the forkhead box (FOX) protein, FoxL2, which is a transcription factor known for its role in folliculogenesis and female sex determination (19). *Foxl2* knockout mice have substantially decreased *Fshb* mRNA and serum FSH levels as well as reduced activin induction of FSHβ (20). Both inhibin and follistatin prevent the stimulatory effects of activin, causing suppression of FSHβ synthesis by blocking activin binding, thereby inhibiting intracellular pathways and subsequent FSHβ transcription (18, 21, 22).

There are several clinical conditions under which FSH expression or signaling via its receptor is increased, resulting in higher circulating levels and ultimately creating a FSH “gain of function” effect. These include ovarian hyperstimulation syndrome (23–26), certain ovarian cancers (27–29), and the recent discovery of non-gonadal FSH actions on bone in transgenic mouse models with elevated human FSH levels (30). GOF effects of FSH in ovaries were also occasionally noted in patients with FSH hypersecreting pituitary adenomas (31). The existence of such conditions highlights the importance of generating animal models that closely mimic human disease phenotypes, allowing us to expand the medical knowledge of these conditions and ultimately providing opportunities to learn how to treat them. While mouse models do not always accurately mimic human pathology, they provide a quick genetic test to address the function of human proteins in a physiological context that cannot be reliably achieved using *in vitro* experimental approaches. In this review, we highlight and describe previously generated FSH gain of function animal models and how they can potentially be used to develop new approaches for treating clinical conditions involving FSH.

### GOF Mouse Models for FSH

Several GOF genetic models have been generated and used to study the physiological consequences of FSH. These models are described below in detail and summarized in Table 1.

**Table 1:** Major phenotypes of FSH gain-of-function genetic models.

**Model**	**Promoter/mutation**	**Major phenotypes**	**Implications**	**References**
10 kb hFSHβ targeted expression (pituitary)	*HFSHB* promoter (Transgenic line)	**Both:** •Sexually dimorphic expression •Gonadectomy resulted in elevated FSH levels in serum, elevated h/m FSHβ mRNA •Treatment with GnRH increases expression 4 to10- fold, which is suppressed by testosterone/ estradiol •Truncation of sequences upstream of 5′ promoter region retained expression of hFSHβ •Truncation of poly-A sequences downstream of 3′ stop codon in exon 3 resulted in complete loss of expression •Replacement of 3′ poly-A sequences with heterologous sequence failed to rescue expression **Males:** •Castration: decreased FSH levels in pituitary •Castrated + testosterone treatment: suppressed mRNA content and serum FSH levels (more so than normal littermates) •Intact: increased testicular weights in adults •Intact: higher serum testosterone levels **Females**: •OVX: increased FSH levels in pituitary •OVX + E_2_ treatment: FSH suppression in pituitary and serum; mRNA suppression •Intact: normal fertility/ litter size/ number of fertilized embryos	Model for study of hFSHβ regulation	(32–35)
Genetic rescue with 10 kb hFSHβ targeted expression (pituitary);	*HFSHB promoter, Fshb null genetic background*. (Type I rescue; FR-I) (Combination of a Transgenic and a knockout)	**Both:** •Targeted expression of FSH in gonadotropes **Males:** •Fertile; restored testes size and structure/ histology, normal sperm count/motility **Females:** •Fertile (10/10), normal litters, corpora lutea (CL) in rescued ovaries readily apparent	Model to study effects of pituitary gonadotrope-targeted expression of FSH on *Fshb* null genetic background	(36, 37)
Ectopic FSH (low)	*mMT-1* promoter (mouse Metallothionein-1); *Fshb* null background (Type II rescue, FR-II) (Combination of a Transgenic and a knockout)	**Both:** •Ectopic expression of FSH **Males:** •Fertile; restored testes size and structure/ histology, normal sperm count/motility **Females:** •Partially fertile (3/10), small litters, small antral follicles and corpora lutea (CL) in rescued 2/3 females died postpartum •Thin uteri, folliculogenesis arrested at pre-antral stage in non-rescued mice •Weak expressors themselves were fertile and had no distinguishable phenotypes from normal littermates	Model to study effects of ectopically expressed FSH	(36)
Ectopic FSH (high)	*mMT-1* promoter (Transgenic line)	**Both:** •Infertile •Elevated serum steroid hormone levels (i.e., testosterone, estradiol, progesterone) **Males:** •Enlarged seminal vesicles, normal testicular size/development •Increased (epididymal) sperm counts •Castration reduced seminal vesicles to size similar to castrated wild-type littermates **Females:** •Large hemorrhagic/cystic ovaries •Fluid-filled translucent ovaries •Some follicles halted at pre-antral stage, some developed normally •Large/cystic kidneys, abnormal kidney development •Die 6–13 weeks due to urinary tract obstruction	Model to study possible role of FSH (and steroid hormones) in human reproductive diseases	(24)
FSH genetic rescue	Ovine FSHβ (*oFshb*) promoter; *Fshb* null background (Combination of a Transgenic and a knockout)	*Double N-glycosylation mutant hFSH compared to WT hFSH* •Low levels in serum (both) •Readily detectable (mutant) FSH levels in pituitary as subunit monomer but not as FSH heterodimer **Males:** •Fertile •Lower testes weights •No rescue with regard to testes phenotypic characteristics (tubule diameter, and sperm counts) **Females:** •Infertile •No estrus cycles •Hypoplastic ovaries and uteri	Model to study possible therapy for FSH ligand deficiency Model to study role of N-glycans on FSH	(36, 38)
Ectopic *HFSHB* Tg+	Rat insulin II promoter (*RIP II*) (Transgenic line) (Combination of a Transgenic and a natural mutant background)	**Both:** •No significant sexual dimorphism in hFSH levels **Males:** •Similar testosterone levels to controls (non-transgenic *hpg*) •No correlation between hFSH and inhibin B levels •Strong positive correlation between hFSH and testis size (at high serum hFSH levels) •Disorganized testes development •Minimal but incomplete spermatogenesis (no fully differentiated spermatozoa) **Females:** •No apparent estradiol response to hFSH •Strong positive correlation between hFSH and inhibin B levels (similar to WT levels) •Strong positive correlation between serum hFSH and ovaries size •Follicle development to type 7 antral follicles, but no corpora lutea found •*hpg* (Tg+ and non-Tg) body weights lower than WT controls at 9–11 weeks •*hpg* Tg+ ovaries 4x weight increase (compared to non-Tg) •Primordial follicle numbers 2 times higher than in WT and *hpg* non-Tg controls •Secondary follicles restored to normal levels (9–11 weeks) •Number of total antral follicles restored to normal levels (9–11 weeks) •Strong positive correlation between inhibin B and antral follicle count •Increased inhibin A expression (compared to non-Tg) •Dose dependent increase in bone mass (*hpg* and non-*hpg*) •Positive correlation between FSH levels and osteoblast/bone surface area •Negative correlation between FSH levels and osteoclast/bone surface area •Ovariectomy of *hpg* mice resulted in: •[-] decrease of serum levels of inhibin A and testosterone •[-] 47% reduction in bone mass •[-] uterine weights similar to control in *non-hpg* Tg mice •Uterine weights increased compared to control in *hpg* (still lower than *non-hpg)* •No detectable *Fshr* mRNA in bone cells •Age-specific decline in litter production in females due to increased embryo-fetal resorptions without affecting the number of ovulations.	Model to study FSH actions alone	(30, 39–41)
FSH re-routed	*HFSHB Mut*; on *Fshb* null background (Combination of a Transgenic and a knockout)	**Both:** •Sexually dimorphic expression •Dense core granule and chaperone proteins co-localized in pituitary with mutant hFSHβ (similar to LHβ) •Secretion of mutant hFSH increased 2–4 times in response to GnRH agonist (no significant release in control mice) •Lower levels of serum LH in mice expressing mutant hFSH (comparable *Lhb* mRNA levels to those in pituitaries of *Lhb ^+/−^* mice) **Males:** •No specific phenotypes **Females:** •Ovarian and uterine morphology similar between mutant and wt hFSHβ expressing mice •Aromatase levels restored to normal in mutant and wt hFSHβ expressing mice at 9 weeks (on *Fshb* null background) •High progesterone levels in mice expressing mutant FSH •6 times more ovulations in mutant FSH (compared to wt hFSH and normal controls) •Identical primordial follicle counts across all groups •Increased pre-antral follicles, CLs, and follicle size in mutant hFSH-expressing mice •Decreased occurrence of atresia in mutant hFSH- expressing mice •Granulosa pro-survival as well as FSH and LH-responsive genes upregulated in mutant hFSH-expressing mice •*Lhb*-null mice not rescued by mutant FSH	Model to study differences in secretion patterns of LH and FSH	(37)
FSH Tg+ in milk	Rat β-casein promoter (Transgenic line) Bovine β-casein promoter (Transgenic line)	**Males:** •No phenotypes reported **Females:** •Recombinant bovine FSH detected exclusively in milk •Larger lumens in mammary glands of β-casein-*hFSH Tg* mice than wild type controls •hFSH detected in milk fluids and epithelial cells of Tg mice and not in controls •Amount of hFSH detected in milk proportional to transgene copy number •hFSH increased cAMP levels in hFSH-R transfected cells with competitive binding (biologically active) •Transgenic platelet count 2 times more that of WT controls •26.7% of highest-expressing line displayed both breast and ovarian granulosa cell tumors with hemorrhagic cysts •Mouse FSH and progesterone levels of Tg mice higher in all phases of estrus cycle than non-Tg littermates	Model to study ectopic expression of FSH in mammary glands	(42) (43)
Pig FSH Tg	Chinese Erhualian Boar FSHα/β promoter + gene including long range cis-regulatory elements (Transgenic line)	**Males:** •Boars: *TG compared to WT controls:* •Serum FSH levels significantly higher •Semen volume, sperm concentration and motility similar •Germ cells per seminiferous tubule increased •Comparable body weights throughout growth •No significant differences in gut microflora or disease markers **Females:** •Mice: *TG compared to WT controls:* •Significant increase in litter number •Significant increase in CL number (at 14–28 weeks) •Increased serum levels of endogenous mouse FSH and estradiol •Decreased serum levels of LH and testosterone- •Decreased LH mRNA content •Boars: *TG compared to WT controls* •Higher serum FSH levels •Higher pituitary FSHβ content •Smaller litter size •Comparable body weights •No significant difference in serum LH and estradiol	Model to study biological effects of pig FSH	(44) (45) (46)
Inhibin-α KO	Inhibin α-subunit gene deletion (Knockout)	**Both:** •Infertile •Gonadal stromal tumors •Increased serum FSH levels •Die from cachexia-like symptoms **Males:** •Testicular enlargement/ hemorrhage •Decrease in number of Leydig cells •Decreased spermatogenesis proportional to tumor size **Females:** •Ovarian hemorrhage •Decreased folliculogenesis proportional to tumor size	Model to understand and study role of Inhibin/ inhibin-α in development as well as its tumor suppressor activity in gonads	(24, 47)
Inhibin-α / FSH double knockout	Inhibin α-subunit and *Fshb* gene deletion (Double knockout)	**Both:** •Delayed body weight loss compared to inhibin single mutants •Less severe cachexia in double mutants compared to mice lacking only inhibin **Males:** •Compared to inhibin single knockouts, double mutants live longer •Testicular tumors in double mutants are less hemorrhagic **Females:** •Compared to inhibin single knockouts, double mutants live longer •Ovarian tumors are less aggressive •Folliculogenesis is not disrupted at early stages but eventually hemorrhagic ovarian tumors develop	Model to study how FSH acts as a modifier factor to regulate gonadal tumors in the absence of inhibin	(24)
FSHR gain of function	Rat androgen binding protein promoter (*rABP*) on *hpg* background (Combination of transgenic and natural mutation) Asp567Gly mutant when compared to *hpg* non-Tg littermates: *TghFSHRwt* *TgD567*G *mutant* *Constitutively active FSHR mutants;* Human *AMH* promoter driving separately expression of *mFshr D580H* or *D580Y* cDNA transgenes; or a *D580Y* knock-in mouse *Fshr* allele *Constitutively active FSHR on Lhr null background*	**Males:** •Fertile (on non-*hpg* background) •Infertile (on *hpg* background) •Testis weights increased nearly 2 times •Treatment with testosterone resulted in larger testis •Testis contained small numbers of both round and elongated spermatids, mature Sertoli cells •Increased number of seminiferous tubules (compared to Tg-FSH group) •Slight rise in serum and significant rise in intra-testicular testosterone levels *Compared to non-Tg hpg littermates* •overexpression: •No effect on testis weight/serum testosterone levels •No additive effect on testis weight with testosterone treatment •No change in expression of steroid synthesis genes •No changes in testis structure/cellular morphology •Treatment with FSH increased cAMP levels 2 times more, basal levels remained the same •No TSH or hCG binding •2 times increase in testis weight •Synergistic effects on testis weight with testosterone treatment •Increased expression of steroid synthesis genes •Later stage spermatogenesis/ post-meiotic elongated spermatids •Treatment with FSH increased cAMP levels (40% as much as *TghFSHwt*), basal levels increased two times more •Binds to TSH and hCG (cAMP levels increased by 40% that of FSH stimulation) •Transgenic *Fshr ^*D*580*H*^* female mice demonstrated hemorrhagic and cystic ovaries, loss of immature follicles, increased granulosa cell proliferation, increased E2 production, unruptured and luteinized follicles and occasional teratomas •Most severely affected transgenic *Fshr ^*D*580*H*^* female mice, in addition, displayed increased prolactin levels and mammary gland hyperplasia, pituitary adenoma formation and adrenal defects •Transgenic and knock-in *Fshr ^*D*580*Y*^* mice showed milder ovarian phenotypes with only hemorrhagic cysts *Compared to WT males* •Fertile •Delayed puberty •Mating trials had lower frequency of pregnancy and litter size •20 times more of *Fshr* mRNA •40% of serum T levels •Normal spermatogenesis and testis/seminal vesicle size •Treatment with antiandrogen had no effect on spermatogenesis or testis size (though reduced seminal vesicle size) while both were arrested in WT **Females:** •fertile (on non-*hpg* background) •No significant differences in ovarian weights between hpg Tg and non-Tg littermates	Model to study downstream pathways involving FSHR signaling	(48, 49) (50) (51)

### Expression of Human *FSHB in vivo*

A transgenic mouse model harboring a 10 kb *HFSHB* transgene was the first mouse model generated to test cell-specific expression of FSH in gonadotrope cells and to identify that species-specific differences exist in FSH regulation (32). The *HFSHB* transgene was cloned into an *EcoR1-Sph1* genomic fragment and microinjected into fertilized one-cell embryos. The resulting transgenic mice exhibit only pituitary-specific *HFSHB* transgene expression, with no ectopic expression in non-pituitary tissues (32). Expression of hFSHβ was found to be localized to only gonadotrope cells in the anterior pituitary gland. The FSH heterodimer presumably incorporated the mouse-α subunit, creating an interspecies hybrid heterodimer with hFSHβ, because no free hFSHβ was detected in serum (32). FSH dimer secretion and pituitary *HFSHB* mRNA expression were higher in both transgenic and normal males than in their female counterparts. The retention of normal gonadotrope-specific expression of FSH and its function in mice expressing hFSHβ demonstrates conservation of regulatory elements and transcription factors for this subunit gene in both mice and humans (32). This mouse model provided a novel approach for studying molecular mechanisms and regulatory elements that are involved in control of the human FSHβ-encoding gene and its expression.

### Gonadal Steroid Regulation of *HFSHB*

The same transgenic mouse model described above was also used for experiments designed to analyze steroid regulation of *HFSHB in vivo* (32, 33). This study included several experimental groups including castrated male and ovariectomized female mice. Castration resulted in elevated serum FSH levels in both normal controls and transgenic males. Similarly, increased serum FSH levels were observed in ovariectomized normal and transgenic females (33). Testosterone replacement after castration in male transgenic mice resulted in suppressed serum FSH levels. Estradiol (E_2_) replacement in ovariectomized females similarly resulted in suppressed serum and tissue FSH content. The sexually dimorphic pattern previously observed, in which both normal and transgenic males exhibiting greater tissue and serum FSH levels than the corresponding females, was also observed in these studies (33). These studies highlight the species-specific differences and suggest that the elements responsible for continued synthesis and secretion of hFSHβ in response to androgens are not present in the mouse pituitary environment (32, 33).

### GnRH-Independent Androgen Inhibition of *HFSHB* Transgene

To further elucidate the direct roles that steroid hormones play in regulation of hFSHβ at the pituitary level, *HFSHB* transgenic mice were used to observe the effect of androgen in the presence or absence of gonadotropin-releasing hormone (GnRH) using both *in vitro* and *in vivo* approaches (34). Since there was an apparent species-specific difference in FSH secretion and molecular mechanisms in response to androgens, GnRH was identified as a possible key regulatory site in the androgen response of human FSHβ. For *in vitro* studies, primary pituitary cultures were obtained from GnRH-deficient *hypogonadal* (*hpg*) mice (9, 52) carrying the *HFSHB* transgene (34). Testosterone treatment in the absence of GnRH resulted in suppression of *HFSHB* mRNA and confirmed the inhibitory action of androgens directly at the pituitary level independent of GnRH (34). *In vivo* experiments in the *hpg HFSHB* mice included daily GnRH injections, which induced *HFSHB* expression in both males and females. Simultaneous administration of testosterone propionate in males completely blocked the stimulatory effect of GnRH, whereas simultaneous E_2_ administration in females only partially inhibited GnRH effects (34). These results demonstrated direct effects of testosterone and E_2_ on hFSHβ subunit expression at the pituitary level as well as an indirect suppression of GnRH as an additional regulatory mechanism (34). Additional hypothalamic site of E_2_ action cannot also be ruled out based on the above data.

Having established that the 10 kb *HFSHB* transgene is appropriately targeted to and hormonally regulated in mouse gonadotropes, a series of deletions were made on the 10 kb *HFSHB* transgene (35). Several independent transgenic lines expressing 5′ and 3′ truncated versions of *HFSHB* transgene were produced and systematically analyzed. These *in vivo* models helped to identify that truncation of sequences upstream of 5′ promoter region retained expression of hFSHβ in mouse gonadotropes, truncation of poly-A sequences downstream of 3′ stop codon in exon 3 resulted in complete loss of expression. Replacement of 3′ poly-A sequences with heterologous sequences (for example, lacZ reporter sequences) similarly failed to confer expression (35).

Since FSH is normally released from the pituitary in response to GnRH, it is of great interest to observe the physiological response to targeted expression of FSH in non-pituitary tissues. Accordingly, mouse models have been generated that drive expression from either specific or multiple ectopic tissues.

### Use of *HFSHB* Transgenes to Achieve Genetic Rescue of *Fshb* Null Mice

An FSH-deficient mouse model was created in 1997 through targeted mutation (*Fshb*^*m*1^*)* in exon 3 of the FSHβ-encoding gene (53). Mice that were homozygous, i.e., *Fshb* null (*Fshb*^*m*1/^ *Fshb*^*m*1^*)*, and therefore FSH-deficient, were generated by intercrossing heterozygous mice. *Fshb* null males displayed decreased testis size, yet were fertile (53). Sperm number was decreased by 75%, however, viability remained unchanged. In contrast, *Fshb* null females with the *Fshb*^*m*1/^ *Fshb*^*m*1^ genotype were infertile, with small ovaries and thin uteri. Ovaries had arrested follicular development at the secondary stage, and lacked any corpora lutea (53).

Genetic rescue of FSH-deficient mice was achieved using two independent methods (36). The type 1 genetic rescue (FR-I) consisted of targeting the previously described 10-kb *HFSHB* transgene specifically to pituitary gonadotrope cells. This genetic strategy resulted in complete rescue of both males and females lacking endogenous *Fshb* (36). Testis size and sperm counts in FR-I males were restored to those observed in wild-type values. Similar results were obtained in FR-1 females, as uterine and ovarian sizes also returned to wild-type values. Normal follicular development, restored estrous cycles, and production of normal litter sizes were observed in FR-I females.

Low- level ectopic expression of *HFSHB* was achieved in multiple tissues using a mouse metallothionein (mMT-1) gene promoter with the goal of genetically restoring reproductive phenotypes in FSH-deficient mice, designated as type 2 rescue (FR-II) (36). The mMT-1 promoter was used to drive ectopic expression of both a hCGα-encoding minigene and a hFSHβ-encoding gene, thereby resulting in expression of hFSH dimer in multiple tissues (36). Male mice expressing ectopic hFSH (FR-II) showed complete restoration of testis size and sperm counts. However, restoration of normal reproductive phenotypes was incomplete in FR-II females. Only 3 out of 10 FR-II females were able to conceive, and litter sizes were small. Arrested folliculogenesis was frequently observed in FR-II females. The small number of FR-II females that were able to become pregnant produced one litter, had obvious corpora lutea, yet small antral follicles (36). The results with the type 2 genetic rescue suggest that ectopic expression of human FSH can completely rescue *Fshb* null male mice, yet only partially rescue *Fshb* null females (36).

### Ectopic Overexpression of HFSH Dimer in Transgenic Mice

Overexpression of FSH may lead to high serum levels and clinical conditions that negatively affect fertility. A transgenic mouse model ectopically expressing human FSHβ using an mMT-1 promoter resulted in male and female mice overexpressing hFSH in several tissues (24). Mice expressing either only transgenic MT-α subunit or only MT-FSHβ were crossed to obtain mice expressing hFSH ectopically (24). Founders expressing hFSH dimer at very high levels were chosen for further analysis. Both males and females were infertile; males showed enlarged seminal vesicles and elevated testosterone levels yet normal testis size and spermatogenesis (24). These high level FSH expressing males were infertile, presumably due to male sexual behavioral deficits secondary to excess testosterone. However, this behavioral phenotype was not tested in these studies. Females displayed arrested folliculogenesis, along with increased serum estrogen, progesterone, and testosterone concentrations. Females also developed urinary tract obstruction and hemorrhagic and cystic ovaries, yet exhibited no signs of tumors (24). Their symptoms were comparable, but not identical to human conditions such as polycystic ovarian and ovarian hyperstimulation syndromes. Most females died between 6 and 13 weeks of age. (24). The overexpression of hFSH in multiple tissues gave insight into these clinical conditions and provided a model that may be used in the future for developing treatments.

### Ectopic Expression of FSH in *hpg* Mice

The role of FSH in gonadal physiology was investigated using a mouse model similar to the models described above that carry a *HFSHB* transgene (39). However, this model expressed transgenic human-FSH (tg-FSH) on a gonadotropin-deficient *hypogonadal (hpg*) background to observe FSH effects independent of LH. The *HFSHB* transgene was cloned into a vector containing the rat insulin II promoter (RIP) and injected into mouse oocytes (39). RIP directed ectopic expression of tg-FSH to the pancreas. Hypogonadism was accomplished by breeding tg-FSH mice to an *hpg* strain containing a truncating mutation that caused GnRH depletion, thereby creating tg-FSH+ *hpg* mice (39, 52). Varying serum tg-FSH levels were found in different strains of mice, allowing for analysis of a range of circulating FSH concentrations. Tg-FSH seemed to have no effect on androgen levels in the tg-FSH+ *hpg* mice, which appeared to be due to underdeveloped epididymis and seminal vesicles. In male tg-FSH+ *hpg* mice, testis size increased as compared to non-tg-FSH *hpg* controls, however, this was only observed in males exhibiting high serum FSH levels (>1 IU/liter). Tg-FSH+ *hpg* female mice secreting high levels of FSH exhibited dose-dependent, elevated inhibin B secretion. Ovaries of tg-FSH+ *hpg* females were also enlarged and exhibited increased follicular development to the antral stage (39).

Additional studies were performed using female tg-FSH+ *hpg* mice to determine the effect of FSH alone on primordial follicle reserve and the role of FSH in early follicular development (40). Partial disruption of follicular development was observed in non-tg *hpg* ovaries. Although development past the primary follicle stage occurred, there were small numbers of early antral follicles. In contrast, tg-FSH+ *hpg* females showed advanced follicular development up to the antral stage, although no corpora lutea were observed in any tg-FSH+ or non-tg FSH *hpg* ovaries due to the absence of LH (40). Significant increases in total primordial and secondary follicle numbers were seen in tg-FSH+ *hpg* females as compared with both non-tg *hpg* and wild-type mice. The total antral follicle count was 15-fold higher in tg-FSH+ *hpg* ovaries than non-tg FSH *hpg* levels, which restored values to wild-type levels (40). The findings from this study indicate an important role of FSH in early follicular development, showing an increase in primordial follicle reserve and stimulation of follicle growth.

Interestingly, when tg-FSH+ female mice alone with progressively rising hFSH levels (2.5–10 IU/ml) were monitored across the life span, age-specific phenotypes were observed. Whereas, tg-FSH+ female mice <22 week of age delivered increased litter sizes, those that were older (>23 week of age) produced decreased litter sizes despite increased ovulations and demonstrated premature infertility due to embryo resorptions and parturition failure. Thus, this model provided a novel *in vivo* scenario in which age-related rise in FSH contributes to female reproductive aging and infertility by a post-implantation defect (embryo-fetal resorption) without directly affecting the ovarian reserve (41).

Contrary to the proposed deleterious and direct effects of FSH on bone osteoclasts in mice (54), ectopic human FSH expression in the above described genetic model caused an increase in bone mass in female mice (30). Similar phenotypes were also observed when the *HFSHB* transgene was expressed on the *hpg* genetic background with a total suppression of endogenous gonadotropins and E2. Expression analysis indicated osteoclasts did not express *Fshr* mRNA and the bone phenotypes manifest only when ovaries were intact. Further studies indicated that bone volume in these transgenic mice positively correlated with ovary-derived inhibin A and androgens. Thus, ectopic human FSH expression in this model suggests FSH acts indirectly to enhance bone function in an ovary-dependent and LH-independent manner (30). The controversy with regard to non-gonadal actions of FSH is ongoing and has been recently described in detail (55).

### Rerouting of FSH Into the LH Secretion Pathway

Transcriptional responses of FSHβ and LHβ encoding genes are different and dependent on GnRH pulse frequencies (10, 11, 56). In immortalized gonadotrope cells, *Fshb* gene transcription is favored by slow GnRH pulses whereas *Lhb* gene transcription is dependent on fast GnRH pulses (10, 11, 56). Once the heterodimers are assembled, FSH is largely released constitutively, while LH is released as pulses via a regulated secretory pathway (10, 11). LH contains a carboxy terminal (C′) heptapeptide that directs its secretion via this regulated pathway. A novel mouse model took advantage of this heptapeptide to observe the physiological response to a mutant FSH that contained this peptide (37). Human transgenes encoding either a wild type (*HFSHB*^*WT*^) or mutant (*HFSHB*^*Mut*^) FSHβ were introduced onto an *Fshb-null* genetic background.

The presence of interspecies heterodimers of mouse-α-and WT or mutant FSHβ subunits in different mouse lines was confirmed by Western blot analysis (37). LH is stored in dense-core granules (DCG) prior to release (57–59). To determine whether mutant FSH was secreted via the same pathway as LH, co-localization of mutant FSHβ subunit and DCG-specific Rab27 was evaluated in gonadotrope cells. Interestingly, the number of mutant FSHβ subunit and Rab27 co-localized gonadotropes was 6 to 8- fold higher than seen in gonadotropes of control mice, where FSH is secreted via the constitutive pathway (37). Co-localization of mutant FSHβ and a chaperone protein chromogranin-A (Chr-A), which is found in the Golgi network in gonadotropes and important for regulated release of LH, was also examined. Co-localization of mutant FSHβ and Chr-A was higher when compared to both control and *Fshb* null mice expressing a *HFSHB*^*WT*^ transgene and was similar to levels seen with co-localization of LHβ (37). These results suggest that the engineered mutant FSHβ (containing the C′ heptapeptide normally only found in LH)- containing FSH heterodimer successfully entered the regulated secretory pathway.

As described above, mice deficient in FSH are infertile, have small ovaries and thin uteri, along with disrupted folliculogenesis (53). *HFSHB*^*Mu*^^t^ transgene was able to genetically rescue *Fshb-null* mice, restoring ovarian and uterine size as well as estrus cycles leading to the presence of antral follicles and corpora lutea in ovaries. Progesterone levels were higher in *HFSHB*^*Mut*^–expressing mice than in control and wild-type mice (37). Interestingly, the number of ovulations was increased from 9 to 10 per cycle in control mice to 55 per cycle in mutant FSH–expressing *Fshb* null mice (37). This was accompanied by the presence of more pre-antral follicles and reduced follicular atresia in ovaries. Mice expressing *HFSHB*^*Mut*^ also demonstrated increased follicle size and enhanced granulosa cell proliferation, leading to a longer reproductive lifespan and follicle survival (37). This mouse model provided a novel approach to studying differential secretory pathways of FSH and LH, and demonstrated a potential role of rerouted FSH in treating age-associated reproductive conditions (37).

### Expression of a Transgene Encoding the Non-glycosylated Human FSHβ Subunit

One of the characteristic features of glycoprotein hormones, including FSH is the presence of N-linked sugar chains on both the α- and β-subunits. FSH possesses four (2 on each subunit) potential N-glycan attachment sites (1–4, 60, 61). The presence or absence of both N-glycans on FSHβ subunit contributes to macroheterogeneity, significantly affects serum half–life and may alter bioactivity (60, 61). To genetically determine the role of N-glycans on the human FSHβ subunit, the nucleotides corresponding to two Thr residues following Asn residues on which N-glycans are added, Asn7 and Asn24, were mutated to Ala, thereby abolishing N-glycosylation events at these two sites (38, 60).

The mutant cDNA transgene encoding the N-glycosylation double mutant FSHβ subunit was targeted to gonadotropes in transgenic mice first and the transgene was subsequently introduced onto *Fshb* null genetic background using the well-established genetic rescue scheme. An *HFSHB* ^*WT*^ transgene similarly was introduced onto *Fshb* null genetic background and the resultant *Fshb* null mice carrying the *HFSHB* ^*WT*^ transgene were used as positive controls (38, 60). Biochemical studies confirmed that the mutant FSHβ subunit inefficiently assembled with the endogenous mouse α-subunit and very little FSH heterodimer was present in pituitary extracts (38). Moreover, media collected from short term pituitary organ culture experiments and serum from mutant FSHβ-expressing *Fshb* null mice showed very low or undetectable levels of FSH by radioimmunoassays. These data indicated that the double N-glycosylation mutant FSHβ subunit was secretion incompetent (38). Moreover, the mutant transgene, unlike the *HFSHB* ^*WT*^ transgene, did not rescue *Fshb* null mice, confirming that even if secreted in low levels, the mutant FSHβ-subunit containing FSH dimer was biologically inactive (38). Thus, these studies provided *in vivo* genetic evidence that N-glycosylation on FSHβ subunit is critical for FSH heterodimer assembly, secretion and action (38).

### Expression of FSH in the Mammary Gland

Ectopic expression of bovine FSH has been achieved in a model targeting its expression in milk secreted from mouse mammary glands (42). This mouse model was created using a rat β-casein gene promoter driving expression of bovine α and FSHβ subunits. The β-casein promoter drives targeted expression in only the mammary gland, making it possible to observe the effects of ectopic bovine FSH expression in a single tissue (42). Transgenic (Tg) mice expressing the transgene were created either by microinjection of both subunits, or by breeding of mice that expressed each one of them separately (42). The presence of tg-FSH was confirmed by northern blot and radioimmunoassay. Bioactivity of tg-FSH was also confirmed by measuring granulosa cell counts and the ability of the cells to produce estrogen (42). The amount of tg-FSH positively correlated with granulosa cell number and estrogen production, and therefore suggesting successful bioactivity of the transgene-derived FSH (42). No other overt phenotypes were observed in transgenic mice. This mouse model provided a novel approach for expressing FSH in mammary glands and releasing it into milk.

A similar model was later generated that also ectopically expressed FSH in mammary glands, but in this case, the transgene encoded human FSH. In these studies, the investigators used the bovine β-casein gene promoter to specifically express human FSH in mammary glands of transgenic mice (43). Milk was collected from tg-hFSH mice to determine FSH concentration using an enzyme-linked immunosorbent assay (ELISA). Two mouse lines showed nearly undetectable levels of hFSH, as compared to one line that had high levels (300 mIU/mL). This variation in hFSH concentration in different lines was most likely due to differing transgene copy numbers (43). *In vitro* biological activity of hFSH was determined by measuring cyclic-AMP (cAMP) production after exposing hFSH receptor-transfected cells to sterilized milk from each cell line. Milk containing hFSH increased cAMP production in the assay, indicating receptor binding and intracellular signaling, and therefore, biological activity of hFSH (43).

Blood cell counts were performed to analyze the effects of any hFSH leakage into the circulation. Several of the transgenic lines showed increased white blood cell and platelet levels as compared to normal mice, red blood cells in the affected animals were also smaller in size (43). Ovarian and breast tumors were observed in one transgenic line, along with collapsed alveoli within the lactating glands. Human FSH also seemed to have a stimulatory effect on endogenous mouse FSH, as mFSH levels were higher at all estrus cycle phases in transgenic mice (43). This unique mouse model displayed distinct physiological responses to ectopic expression of hFSH in mammary glands and secretion into milk. Many of them negatively affected blood circulation and reproductive health. Ectopic hFSH leaking into the bloodstream appeared to lead to overproduction of endogenous mouse FSH, possibly contributing to the observed ovarian and breast tumors by increased estrogen production as a secondary consequence (43).

### Expression of Porcine FSH in Mice

Transgenic expression of porcine FSH using bacterial artificial chromosome (BAC) methods has been achieved using a gain-of-function mouse model (45). A BAC containing porcine (p) α and β subunits was constructed and isolated from a porcine BAC library from a male Erhualian pig, a highly prolific pig breed. BAC clones containing pFSH α and β were then digested and microinjected into fertilized mouse zygotes (45). Transgenic (Tg) mice expressing pFSH were identified by PCR and Southern blot. After further breeding of Tg mice to wild-type mice, both pFSHβ and pFSHα BACs were transmitted in identical Mendelian ratios to offspring, indicating proper hybridization of the Tg subunits. Expression of Tg pFSH mRNA was confirmed by RT-PCR and northern blot, with expression localized specifically to the pituitary gland. Circulating pFSH was confirmed by evaluation of serum samples by ELISA, showing levels ranging from 6.36 to 19.83 IU/L, which were within the physiological range (45).

Female fecundity was analyzed in both Tg and WT mice. Interestingly, Tg females had litter sizes that were significantly larger than WT females as well as the total number of pups than WT littermates. This increase in fecundity in Tg females seemed to be due to enhanced ovulation, as histological examination revealed a significant increase in the number of corpora lutea at 14 and 28 weeks of age in Tg mice compared to WT. Serum hormone levels were analyzed to determine if increased ovulation was due to differential hormonal regulation (45).

Endogenous mouse FSH levels were elevated in Tg mice, as was estradiol. Serum levels of LH and testosterone were significantly lower in Tg mice compared to WT. Pituitary expression of LHβ mRNA was also lower in Tg mice than in WT mice (45). The increased estradiol levels could be due to greater aromatization of androgens to estrogens due to elevated levels of endogenous mFSH in addition to transgene-derived pFSH (45). This enhanced conversion of testosterone to estrogen as a result of increased aromatase activity could explain the lower serum levels of testosterone. However, the mechanism for reduced LHβ mRNA and serum LH despite low levels of serum testosterone is unclear. The results from this study provided confirmation of successful expression of porcine FSH in a transgenic mouse model with no reproductive effects in males but enhanced ovulation in female Tg mice (45).

### Expression of Transgenic Porcine FSH in Large White Boars

Porcine FSH was further analyzed by the same group that produced BAC pFSH subunits from Chinese Erhualian pigs, and introduced them into Large White Boars (44). As the Large White Boar previously showed poor reproductive performance, the investigators sought to determine if overexpression of pFSH from a highly prolific pig breed could improve fertility. Successful integration of pFSH into transgenic (Tg) boars was confirmed using genomic PCR as well as RT-qPCR analysis to determine mRNA expression of porcine FSHα and FSHβ. Expression of FSHα was observed in multiple tissues including heart, liver, spleen, kidney, brain, testis, and epididymis of both Tg and wild type (WT) boars, with higher expression in Tg than WT.

Expression of FSHβ was localized specifically to the pituitary and was significantly higher in Tg than WT boars (44). Higher serum levels of both FSHα and FSHβ were observed in Tg boars suggesting overexpression of pFSH. Male reproductive performance was measured by evaluating semen volume, sperm motility, sperm concentration, and total sperm number per ejaculation (44). There was no significant difference between semen quality parameters of Tg and WT boars. However, the number of germ cells per seminiferous tubule was significantly higher in Tg boars than WT (44). The elevated germ cell counts in seminiferous tubules suggested increased spermatogenesis capacity, but the lack of significant results from semen evaluation leaves room for further analysis to confirm this possibility (44).

In a third study done using the same BAC system containing pFSH α and β, analysis of reproductive phenotypes in female transgenic (Tg) Large White Boars were analyzed. Methods for pFSH BAC transfer were the same as previously described for this model (44). The specific integration site of pFSH into Large White Boars was determined using whole-genome sequencing, identifying exogenous FSHα/β genes at 140, 646, 456 bp on chromosome 9 (46). The transgene integration site was mapped to perhaps rule out that the integration itself did not result in modification of any endogenous loci that regulate fertility.

Analysis of Tg female boars revealed elevated levels of serum FSH and FSHβ protein in the pituitary, but Tg females produced reduced numbers of total newborn piglets as compared to WT. Reduced expression of *Fshr, Lhr, Esr1*, and *Esr2* was also observed using RT-qPCR in Tg boars as compared to WT at 300 days of age. Reproductive organ weights, blood cell counts, and histological analysis revealed no differences between Tg and WT boars in overall reproductive health (46). The reduced expression of mRNAs encoding receptors for FSH, LH, and estrogen suggest a possible negative effect of pFSH overexpression in female pigs as well as the observation of reduced total newborn piglets (46). Further studies using this model are needed to confirm the effects. However, these studies provided novel information on the physiological role of porcine FSH *in vivo* in the homologous species.

### Inhibin Knock Out Mouse Model

Important regulators of FSH production and secretion are gonad-derived dimeric growth factors, activin and inhibin. Inhibin is a heterodimer, consisting of an α and β subunit, whereas activin is a homodimer, consisting of various combinations of the two homologous β subunits (βA and βB) (21). The inhibin and activin subunits are expressed in multiple tissues throughout the body. Inhibins suppress and whereas activins promote FSH synthesis and secretion. To investigate the role of inhibin in reproduction and general physiology, a knockout mouse model with a targeted deletion of the α-inhibin–encoding gene was achieved using homologous recombination technology in mouse embryonic stem cells (47). Targeted deletion of only the α-subunit of inhibin ensured successful inhibin deficiency without unwanted deletion of the activins.

Male and female heterozygous mice produced normal litter sizes and were fertile. However, homozygous males and females proved to be infertile when crossed with wild-type mice, despite developing normal external genitalia (47). In addition to infertility, all homozygous mice tested showed gonadal tumors when examined histologically, which were evident as early as 4 weeks of age. Testicular enlargement was visible in males, along with gradual regression of spermatogenesis and a decrease in Leydig cell count starting at 5–7 weeks of age (47). In females, ovarian tumors disrupted follicular development and morphology from 7 to 16 weeks of age. FSH levels in both males and female homozygous, inhibin-deficient mice were 2- to 3-fold higher when compared to both heterozygous and WT control mice (47). The results of this study suggested a novel secreted tumor suppressor role for inhibin that was highly specific to the gonads. Development of normal external sexual organs and gametes followed by regression and disruption at a later age indicated normal embryogenesis and early sexual development, therefore suggesting gonadal tumors as the cause for infertility (47).

To further investigate the role of inhibin and FSH in gonadal tumorigenesis, a double-mutant knockout approach was taken. Since the inhibin-deficient mice developed aggressive gonadal tumors accompanied by elevated FSH levels, the contribution of FSH to tumorigenesis was directly assessed using a genetic strategy. To achieve this, double-homozygous mutant mice were created by intercrossing heterozygotes for each knock-out mutation (*Inha*^*m*1^ and *Fshb*^*m*1^) to generate mice deficient in both inhibin and FSH (*Inha*^*m*1^*/Inha*^*m*1^*; Fshb*^*m*1^*/Fshb*^*m*1^) (24). The first parameter examined in the double-knockout mice was weight, as the previous study showed that mice deficient in inhibin only, exhibit a severe cachexia-like syndrome and die by 12 weeks of age (24). Most of double-knockout mutant males survived for 1 year and showed no dramatic weight loss or testis phenotypes. Approximately, 95% of the inhibin-deficient female mice died by 17 weeks. In contrast, double null mutant females, about 70% survived past 17 weeks, but these all eventually died by 39 weeks and all of them exhibited severe weight loss (24).

Gonadal tumor progression was also altered in the double-mutant mice, as development of tumors was slower and less aggressive than in mice deficient in inhibin alone (24). In 12 week old double mutant males, the tumors were small, there were no signs of hemorrhage, and tubule morphology was also unaltered by tumor growth, despite proliferation of tumor cells (24). Beyond 1 year of age, some males showed no tumor development, as compared to inhibin-deficient males which all had tumors as early as 4 weeks (24).

In female double-mutants, ovaries appeared morphologically normal at 12 weeks of age. Histological analysis revealed hemorrhage, cysts, as well as granulosa cell tumors. However, these tumors in the double-knockout females appeared less invasive and developed more slowly than in inhibin-deficient mice. Both male and female double-mutant mice showed reduced serum levels of activin A and estradiol as compared to mice lacking only inhibin (24). In addition to this, aromatase mRNA expression levels were reduced in double-mutant mice compared to those in inhibin-deficient mice. These results confirm the role of inhibin in gonadal tumorigenesis and identify FSH as an important modifier in the progression and aggressive growth of inhibin-deficient gonadal tumors (24).

### FSH Receptor Gain of Function

The FSH receptor (FSHR) is a transmembrane, G-protein coupled receptor expressed on testicular Sertoli cells and ovarian granulosa cells in males and females, respectively (2, 4). Signaling via FSHRs results in steroidogenesis (production of estrogen) and is essential for gonadal development in both sexes. A novel approach to studying the gain-of-function effects of FSH receptor was undertaken by generating a mouse model exhibiting constitutively active FSH receptor action on an *hpg* genetic background (48). The use of the *hpg* genetic background allowed observation of the effects of active FSHR completely independent of endogenous gonadotropins, FSH and LH.

The gain of function receptor mutation (*FSHR*+) was a single amino acid substitution (Asp567Gly) that was specifically expressed in Sertoli cells by using the rat androgen binding protein (rABP) promoter (48). Bioactivity of the ligand-independent FSH receptor was confirmed by measuring cAMP production, which was significantly higher in Tg-Sertoli cells than in non-Tg-*hpg* Sertoli cells *in vitro* (48). The *in vivo* effects of the *FSHR*+ mutation were first examined by measuring testicular weight. *Tg-FSHR*+ *hpg* testis weights were increased up to 5-fold, with an average of a 2-fold increase as compared to non-Tg *hpg* controls. Histological examination of *FSHR*+ testes showed round and elongated spermatids and signs of Sertoli cell maturation, as compared with non-*FSHR*+ *hpg* controls that lacked mature Sertoli cells and exhibited blocked spermatogenesis. Tg-*FSHR*+ mice also had elevated testosterone levels, and undetectable levels of LH and FSH, suggesting that the observed physiological responses to the constitutively active FSH receptor was indeed gonadotropin-independent (48).

To determine whether the results from the *FSHR*+ mutation were solely due to the amino-acid substitution or if they were in response to overexpression of mutant FSH receptor, a comparative study was performed using the same mouse model in parallel with a transgenically overexpressed wild-type human FSH receptor (*TgFSHRwt*) (49). Transgenic male and female mice overexpressing *TgFSHRwt* were created by microinjection of a human *FSHR* DNA construct into mouse oocytes. Overexpression of *TgFSHRwt* was confirmed by measuring radioactive ^125^I-FSH binding to testicular membranes. Significantly elevated ^125^I-FSH binding was observed in *TgFSHRwt* testes as compared to non-Tg controls, similar to levels in *FSHR*+ mice, thereby confirming overexpression.

*TgFSHRwt* mice displayed no difference in testis weights or serum testosterone (T) levels, as compared to *FSHR*+ mice, which had larger average testis weights and elevated T concentrations when compared to non-Tg controls. *TgFSHRwt* Sertoli cells showed higher cAMP activity *in vitro*, however basal levels did not exhibit the same increased activity as *FSHR*+ cells (49). Receptor ligand-specificity was decreased in *FSHR*+ mice, as exposure to human chorionic gonadotropin and TSH resulted in intracellular signaling. However, the same response was not observed in *TgFSHRwt* mice. Steroidogenic enzyme-encoding mRNAs such as *Cyp11a1* and *Star*, were elevated in *FSHR*+ mice, suggesting increased steroidogenic potential (49). However, this increase was also not observed in *TgFSHRwt* mice. Sertoli and germ cell maturation that was observed in *FSHR*+ mice was also absent in *TgFSHRwt* mice, as they exhibited immature development similar to non-Tg *hpg* mice. Together, all of these results suggest that the physiological responses to constitutively active mutant FSH receptors in *FSHR*+ mice were due to the mutation itself and not the result of receptor overexpression (49).

Activating mutations in human *FSHR* are very rare. To identify the phenotypic consequences of such mutations in humans, mouse models harboring mutant versions of *Fshr* were first developed (26, 50). The rationale was that phenotypic analysis of these mice would provide information to look for similar phenotypes in patients carrying analogous mutations. Two independent *Fshr* point mutants D580H and D580Y were created and expressed using human *AMH* promoter to achieve ovary-specific expression in transgenic mice. Additionally, an *Fshr* BAC clone was engineered to carry the D580Y mutation and knocked-in to the endogenous *Fshr* locus in ES cells first and subsequently, the knock-in mutant mice were generated (26, 50).

Both D580H and D580Y mutants displayed increased basal receptor activity and they both demonstrated FSH binding. D580H mutant FSHR was neither ligand-dependent nor promiscuous toward LH/CG stimulation (26, 50). Granulosa cell-specific expression of *mFshr* ^*D*580*H*^ resulted in multiple ovarian abnormalities in transgenic female mice. Ovaries in the majority of transgenic females displayed hemorrhagic cysts, accelerated loss of immature follicles, increased granulosa cell proliferation and E2 biosynthesis, and luteinized but unruptured follicles, and teratomas (26, 50). A subset of the most severely affected transgenic females were infertile due to disrupted estrus cycles, and decreased gonadotropin, and increased prolactin levels. The increase in E2 and PRL levels led to secondary abnormalities including mammary gland enlargement and hyperplasia, pituitary adenoma formation, and defects in adrenal glands (26, 50). In contrast to phenotypic consequences of *mFshr* ^*D*580*H*^ expression, either transgenic or knock-in expression of *mFshr* ^*D*580*Y*^ mutant resulted in milder phenotypes, mostly hemorrhagic cysts in ovaries (26, 50). Thus, these GOF *Fshr* mutant mice resulted in distinct changes in ovarian function and proved valuable in the search of similar mutations in humans.

The most recent FSH receptor gain of function model challenged the traditional dogma that testosterone is essential for spermatogenesis (51). A mouse model was created that possessed a constitutively active mutant FSH receptor on an LH receptor null background (*Fshr-CAM/Lhr*^−/−^) (51). As LH regulates testosterone production via binding of LH receptors (LHRs) in Leydig cells, it was hypothesized that an *Lhr* knockout approach would eliminate testosterone action. However, testosterone production persisted in the initial *Fshr-CAM*/*Lhr*^−/−^ male mice, as serum levels recovered to about 40% of wild-type concentrations. Therefore, the rescue of testicular size and spermatogenesis was probably due to normal testosterone actions (51). To eliminate any basal T activity, a treatment using the antiandrogen, flutamide, was employed. In WT control mice that had no *Fshr-CAM*, reduction in the size of testes and seminal vesicles was observed as well as arrested spermatogenesis. Interestingly, the *Fshr-CAM*/*Lhr*^−/−^ males had only reduced seminal vesicle size after anti-androgen treatment, with no change in testicular size, and normal spermatogenesis (51). In addition to this, expression of androgen-dependent genes (*Drd4, Rhox 5, Aqp8*, and *Eppin)* were tested in the *Fshr-CAM*/*Lhr*^−/−^ males. Anti-androgen treated WT males showed reduced expression profiles of these genes, whereas mutant *Fshr-CAM*/*Lhr*^−/−^ showed no reduction (51). These results suggested that even in the absence of testosterone, constitutively active FSH receptor alone is able to maintain androgen-dependent gene expression as well as normal spermatogenesis and testicular development.

## Conclusions and Future Directions

**Figure 1:**
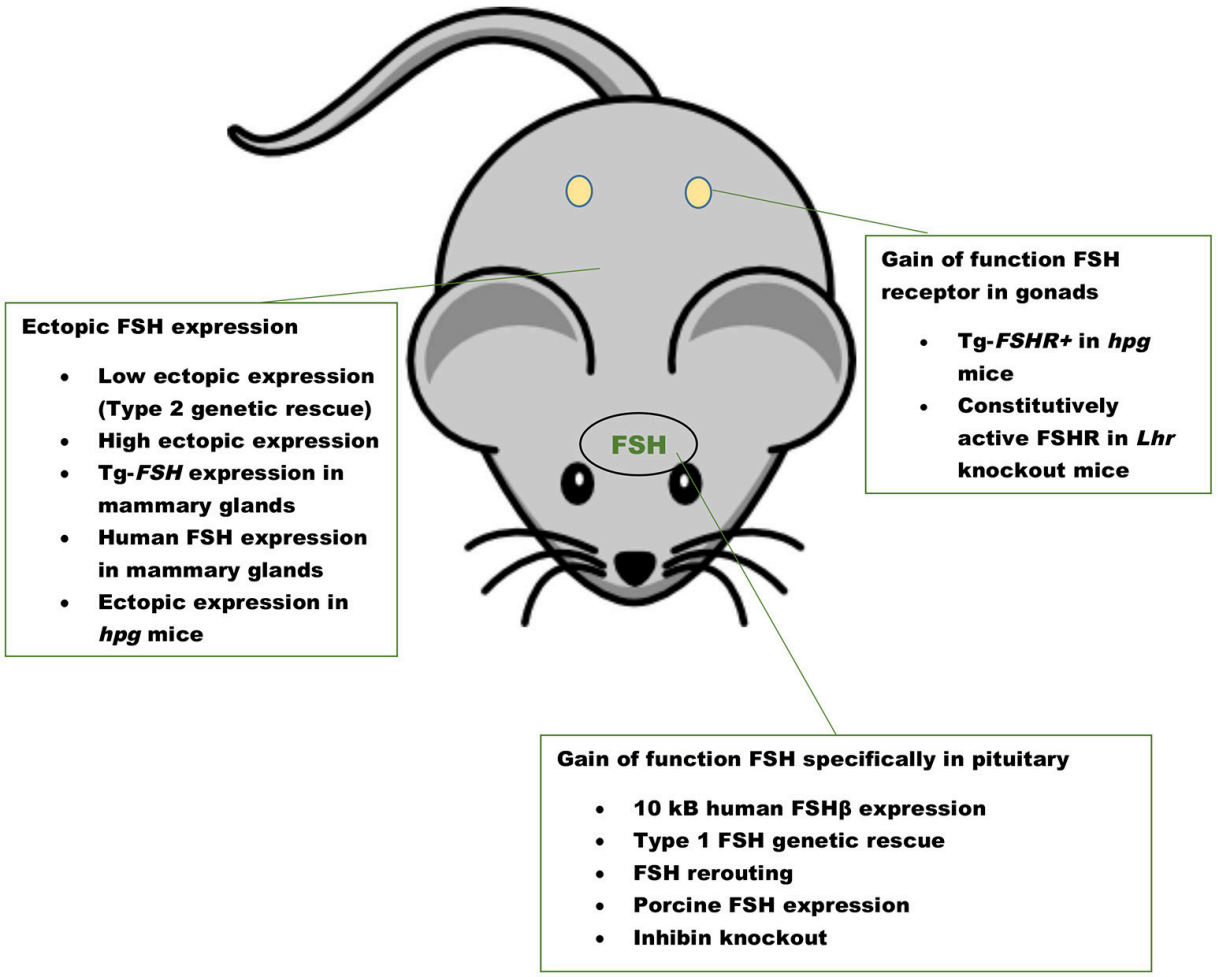
Gain of function mouse models for FSH ligand and FSH receptor. A summary of mouse models with pituitary-targeted and ectopic expression of FSH and gain of function mouse models for FSHR activation. Inhibin knockout mice have high levels of FSH as a result of loss suppression by inhibin.

**Figure 2:**
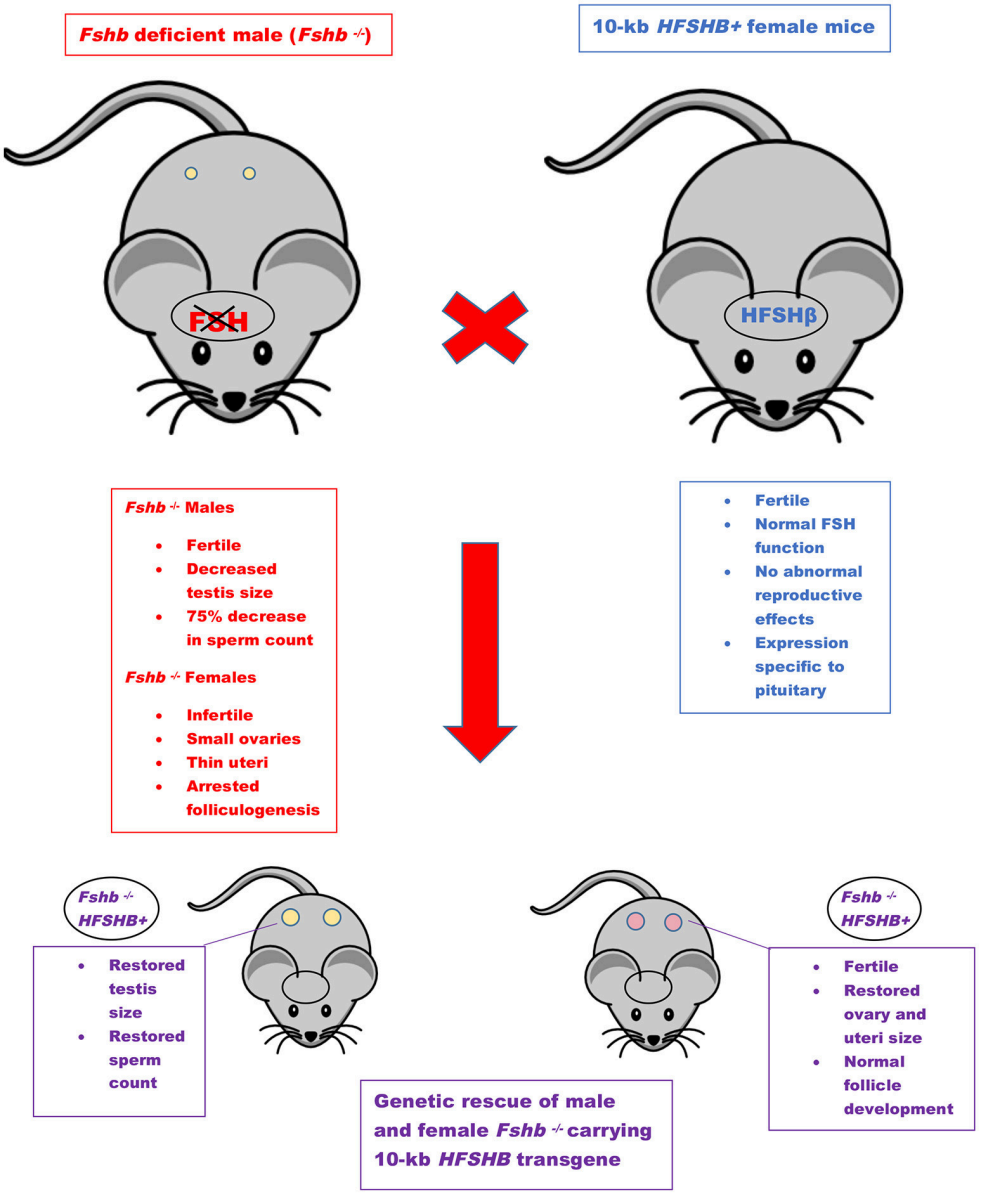
Genetic rescue scheme. Mice lacking *Fshb* show sexually dimorphic phenotypes (red box). The features of *HFSHB*+ mice were shown in blue box. *HFSHB*+ mice by themselves do not exhibit any overt phenotypes. The *Fshb* null male mice are typically intercrossed with *HFSHB*+ transgenic female mice to eventually generate *Fshb*^−/−^ *HFSHB*^+^ mice. The *HFSHB*^+^ transgene fully rescues both male and female *Fshb* null mice (purple boxes).

The first GOF mouse model for FSH was generated by our group more than 25 years ago (32). Since then investigators have used different promoters to achieve gonadotrope-specific as well as ectopic expression of the FSH ligand. Most commonly, human *FSHB* gene or individual subunit-encoding cDNAs (*CGA* or *FSHB*) were used in these experiments. More recently, BACS, or genes encoding porcine FSHβ subunit were also used to create transgenic pigs. GOF models for FSH action were generated using constitutively active FSH receptor–expressing mice and the reproductive consequences were studied either in these models directly or on an *Lhr* null genetic background (Figure 1). Combination of *Fshb* null mice and FSH GOF models resulted in genetic rescue that was used as an efficient *in vivo* functional assay for testing bioactivities of FSH and FSH analogs (Figure 2). A summary of the major phenotypes observed in each model is listed in Table 1.

Promoters used to generate FSH GOF models have also proved useful to express different reporters specifically in the gonadotrope cell lineage (62, 63). Recent advances in temporally regulated gene expression (64–66) will allow us in the future to tightly control FSH expression in desired tissues at desirable times across the life span of a mouse. Such refined genetic models will be useful to identify age-dependent gene/protein networks in FSH target tissues. These genetic models to conditionally “turning on” FSH in desired cells will also allow us to test if FSH receptor expression in non-gonadal cells has any physiological significance (55).

## Author Contributions

RM wrote the first draft of the manuscript and created the Figures. CS generated the entire Table. TK edited the manuscript, Table, and Figures. He also wrote the abstract, about Glycosylation, and Conclusions, and Future Directions. He created and formatted the entire Bibliography.

### Conflict of Interest Statement

The authors declare that the research was conducted in the absence of any commercial or financial relationships that could be construed as a potential conflict of interest.

## Abbreviations

BAC: Bacterial Artificial Chromosome
cAMP: 3′,5′-cyclic adenosine monophosphate
DCG: Dense Core Granules
E2: Estradiol
FSH: Follicle-stimulating hormone
FSHR: FSH-receptor
FoxL2: Forkhead box L2
FR-I: *Fshb* type - I genetic rescue
FR-II: *Fshb* type - II genetic rescue
GnRH: Gonadotropin releasing hormone
GOF: Gain-of-function
GPCR: G-protein Coupled Receptor
hCG: Human chorionic gonadotropin
HPG: Hypothalamus-Pituitary-Gonadal
*hpg*: Hypogonadal
LH: Luteinizing hormone
LHR: Luteinizing hormone receptor
mMT-1: mouse metallothionein- 1
RIP: Rat insulin II promoter
RT-PCR: Reverse transcription- polymerase chain reaction
T: Testosterone
Tg: Transgenic
TSH: Thyroid-stimulating hormone
m: mouse
h: human
p: porcine
o: ovine
WT: Wild-type.
